# Multi-membrane search algorithm

**DOI:** 10.1371/journal.pone.0260512

**Published:** 2021-12-06

**Authors:** Qi Song, Yourui Huang, Wenhao Lai, Tao Han, Shanyong XU, Xue Rong

**Affiliations:** 1 School of Electrical and Information Engineering, Anhui University of Science and Technology, Huainan, China; 2 Anhui Science and Technology University, Chuzhou, China; Northeast Electric Power University, CHINA

## Abstract

This research proposes a new multi-membrane search algorithm (MSA) based on cell biological behavior. Cell secretion protein behavior and cell division and fusion strategy are the main inspirations for the algorithm. In order to verify the performance of the algorithm, we used 19 benchmark functions to compare the MSA test results with MVO, GWO, MFO and ALO. The number of iterations of each algorithm on each benchmark function is 100, the population number is 10, and the running is repeated 50 times, and the average and standard deviation of the results are recorded. Tests show that the MSA is competitive in unimodal benchmark functions and multi-modal benchmark functions, and the results in composite benchmark functions are all superior to MVO, MFO, ALO, and GWO algorithms. This paper also uses MSA to solve two classic engineering problems: welded beam design and pressure vessel design. The result of welded beam design is 1.7252, and the result of pressure vessel design is 5887.7052, which is better than other comparison algorithms. Statistical experiments show that MSA is a high-performance algorithm that is competitive in unimodal and multimodal functions, and its performance in compound functions is significantly better than MVO, MFO, ALO, and GWO algorithms.

## 1. Introduction

In the past few decades, based on linear and non-linear programming methods optimization algorithms have been used to solve various practical problems in engineering, science, business, economics, etc. These methods may require larger gradient information, and usually need to improve the solution near the starting point [1]. However, with the continuous expansion of artificial intelligence applications, in the research of optimization problems, the traveling salesman problem, assignment problem, and workshop scheduling problem are beyond the capabilities of traditional optimization algorithms. These engineering problems are highly non-linear, including complex objective functions with a large number of different variables which are usually subject to many constraints [2]. Compared with traditional optimization techniques, meta-heuristic algorithms are more suitable for solving practical problems with unknown derivative information [3]. It is because meta-heuristic algorithms have good random search capabilities. This mechanism avoids the stagnation of local optimal solutions. Therefore, the meta-heuristic algorithm provides new solutions to some complex problems, which has proved to be a successful idea [4].

Regardless of the different sources of inspiration for the group meta-heuristic al-gorithm, the search process is in two stages: exploration and development [5]. In the exploration phase, the algorithm will continue to randomly explore the global area to expand the search area as much as possible [6]. In the development stage, the algorithm is based on the global search results and performs a local search for the are-as where the optimal solution may exist. Different algorithms use their own search strategies, but whether they can maintain a balance between exploration and development is an important criterion for optimization capabilities. The literature [7] maintains the balance by increasing the diversity of candidate solutions, and the literature [8] adjusts the mutation ratio of the algorithm. In addition, different algorithms can be combined to obtain better balance ability [9, 10].

Inspired by cell membranes [11], the idea of membrane computing has first proposed by Professor GP, an academician of the Romanian Academy of Sciences, in 1998. The essence of membrane computing is to abstract the different functional organs of cells into membrane functions, to realize the capability of computing like cells. By learning and simulating the way cells, tissues, organs or other biological structures process chemical substances, a distributed computing model with good computing capabilities is established. However, technological advances in various fields of engineering and science have led to many challenging real-world problems [12]. Such as: pressure vessel design, welded beam design, and many other engineering problems with equality and inequality constraints [13, 14]. Different meta-heuristic algorithms show powerful computing power on various problems [15]. The fact has proved that based on the "No Free Lunch" (NFL) theorem [16], there is no universal optimization algorithm inspired by nature that can solve all real-world optimization problems in the best way [17]. It means that a certain type of membrane calculation is suitable for solving a specific set of problems, but it cannot effectively solve all types of problems.

This work has proposed the multi-membrane search algorithm (MSA), for solving constrained and global optimization problems. Cells usually have strong adaptability when the environment changes [18]. We regard the function to be solved as the fitness function of the algorithm. In the optimization process, the group individuals imitate the behavior of cell production of protein, and the resulting multi-dimensional solution is used as the candidate solution generated by the algorithm as a function.

## 2. Multi-membrane search algorithm introduced

### 2.1 Inspiration

**Fig 1:**
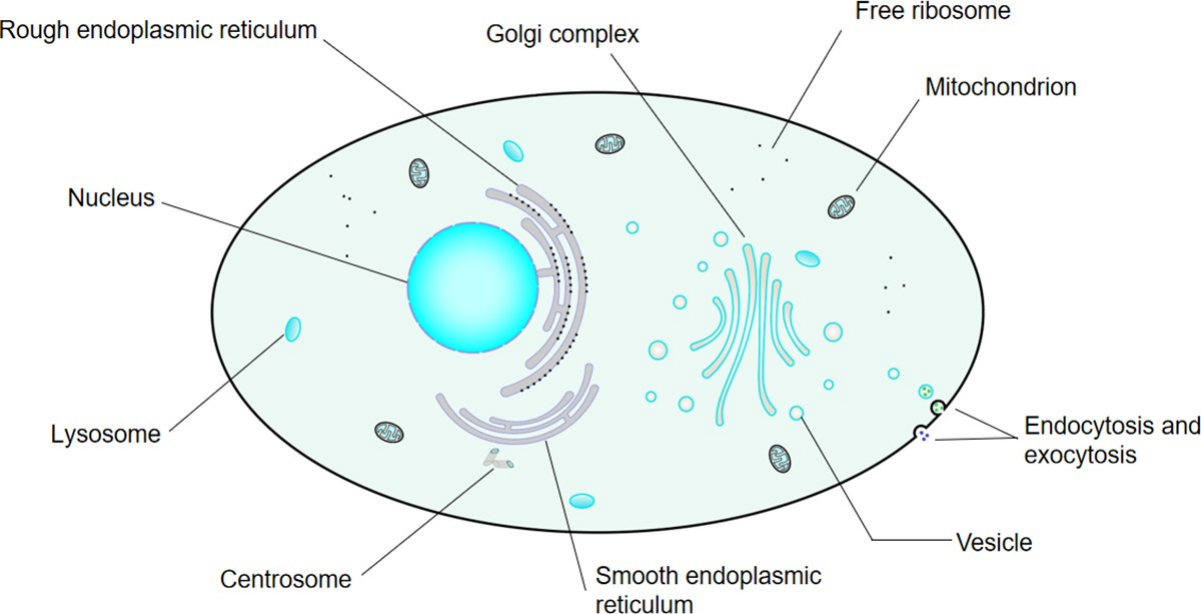
Cell model.

As shown in Fig 1, the basic function of biofilm is to partition. We call the outer cell membrane the basic cell membrane, which is the basic operating unit of the multi-cell membrane element heuristic algorithm. We call the intracellular organ membrane the sub-membrane. The different transcription of RNA by the sub-membrane is the cause of individual differences in cells.

We abstract the functions of various organelles in the cell as sub-membrane structures. Different sub-membranes have different functions to mimic the functions of the organelles. The optimal solution of the previous step is processed differently through different sub-membranes, and each cell can choose between the new results produced by its sub-membrane. Information exchange is carried out through the way of cell mating and reproduction, and finally, highly parallel computing is realized.

### 2.2 Mathematical models

#### 2.2.1 Mathematical model of cell population

Most group algorithms divide the search process into two parts: exploration and development. We use the nucleus of a single cell to guide the organelles to explore the process of intracellular protein synthesis and realize the development process of cells approaching the optimal individual through cell division and fusion.

The MSA is introduced as follows:

$$M=\{C_{1}\;C_{2}\;\cdots \;{C_{i}\;\cdots \;C}_{I}\},$$ (1)

Among:

*M* represents the MSA group optimization algorithm;

*I* Represents the number of individual cells;

*C*_*i*_ Represents the *i*-th cell in the MSA.

In this paper, *I* is set to 10, which means that each iteration of the MSA has a total of ten cells to perform operations.

#### 2.2.2 Single cell membrane model

The single-cell membrane system generally uses characters or strings as objects, which is different from the binary encoding of the classic genetic algorithm, and the MSA uses decimal encoding. Every cell has organelles used to synthesize proteins. The functions of organelles are different. We abstract organelles as sub-membranes in the single- cell membrane system of the MSA. In this way, different membranes have their own set of rules, and due to the role of the membrane, these rule sets do not affect other sub-membrane objects.

*C*_*i*_ structure is as follows:

$$C_{i}={\left[\begin{array}{c} X_{1}\\ X_{2}\\ \vdots \\ X_{j}\\ \vdots \\ X_{J} \end{array}\right]}_{i}$$ (2)

Among:

*X*_*j*_ represents the *j*-th organelle submembrane in *C*_*i*_;

*J* represents the number of organelle membranes in a single cell, and is also the number of candidate solutions included in the single cell membrane system, and *J* = *K*+1.

#### 2.2.3 Organelle model

**Fig 2:**
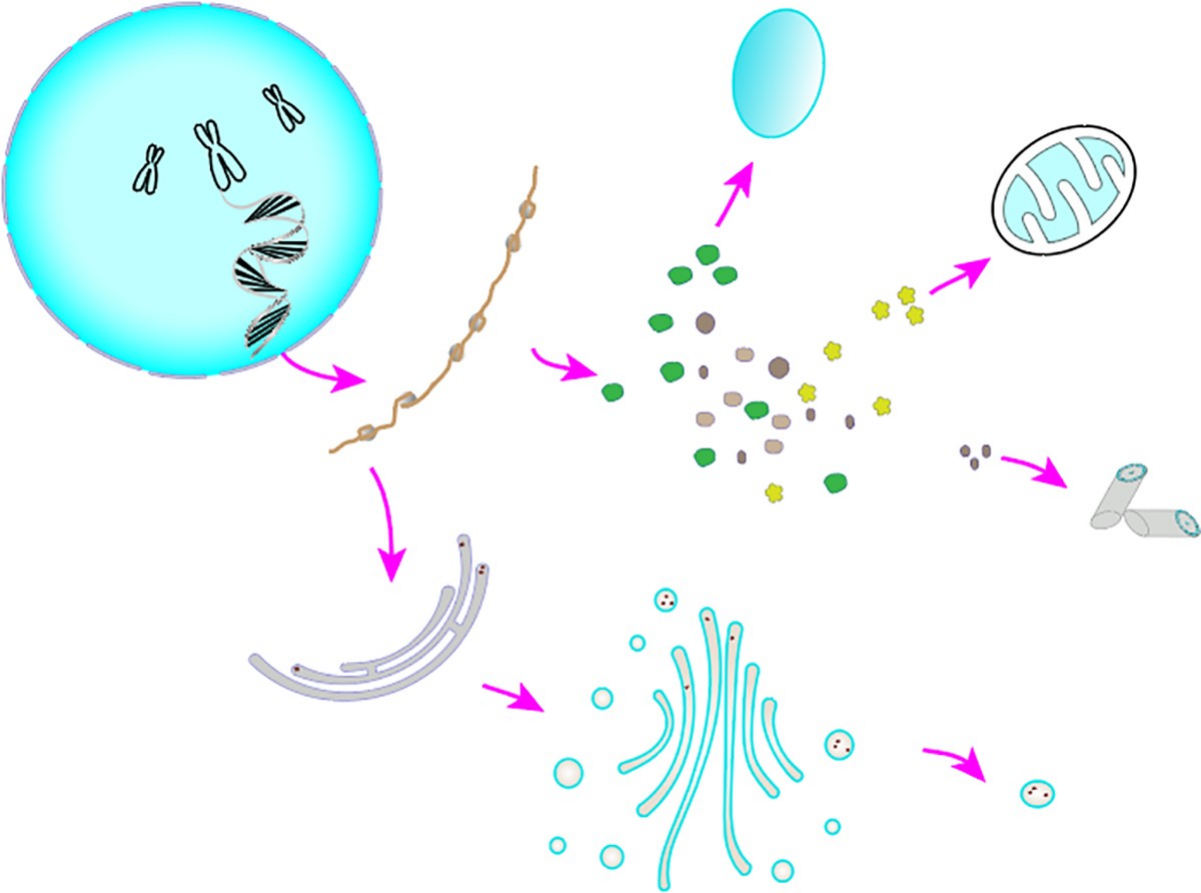
Cell synthesis protein.

As shown in Fig 2, the one organelle is often only responsible for one type of function. For a submembrane, it only processes its own functions. For the MSA to optimize the multi-objective optimization function, for each *C*_*i*_, the real number combination of the initial randomly generated array is used as the feasible solution of the function to be optimized, and the individual with the optimal solution in these object combinations is used as the RNA transcribed from the nucleus. We call the sub-membrane where the optimal solution is located in the current step size as the nuclear membrane of the current system *C*_*i*_, denoted by *x*^*best*^. Similar to cell life activities, different organelles can process proteins of different properties based on the same RNA. The *x*^*best*^ membrane outputs the optimal solution as RNA, and each of the remaining sub-membranes uses the optimal solution as the processing basis.

**Fig 3:**
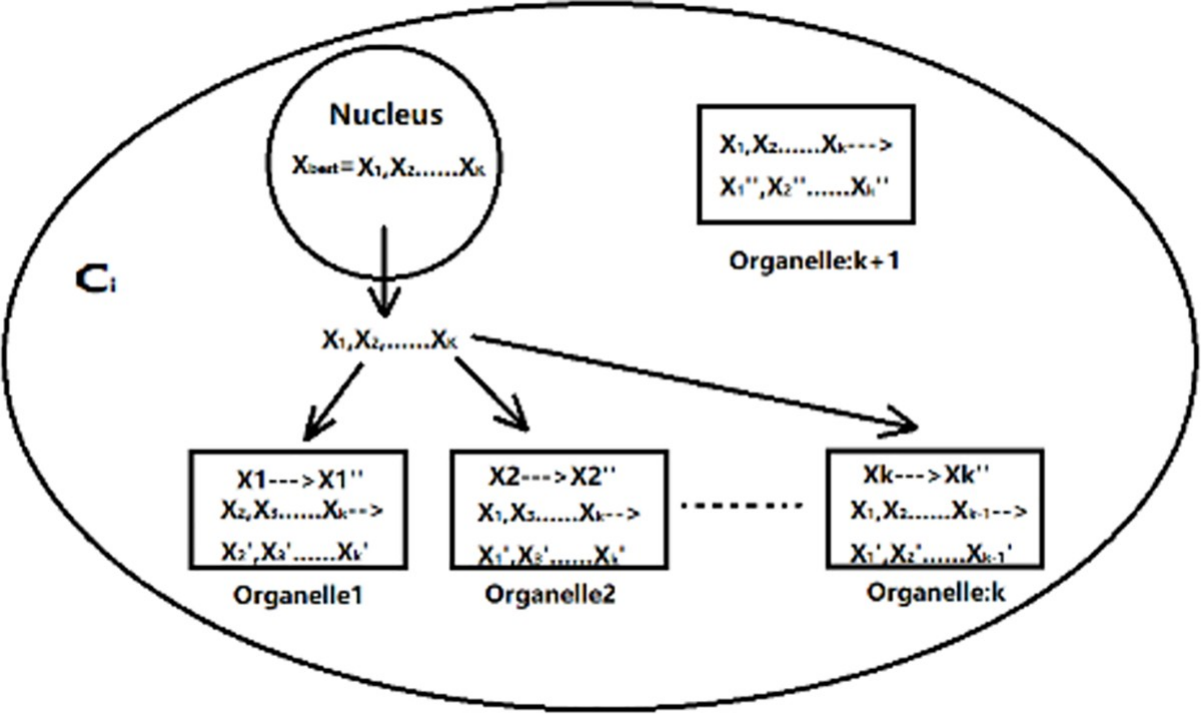
Simplified diagram of the new solution model of MSA cell individual generation.

As shown in Fig 3, each sub-membrane only rewrites the dimension of the optimal solution related to its function, and gradually approaches the optimal solution of the current single-cell system for other dimensions, thereby generating new solutions. Therefore, the dimension of the solution is the same as the number of functional organelles. At the same time, an additional sub-membrane with collection rules is provided in the cell membrane, which can summarize all the functions of rewriting information. Therefore, a single cell membrane system contains a total of *K*+1 sub-membrane. After the processing of each sub-membrane is completed, the obtained results are compared with the original optimal results to realize the function of cell optimization.

For the organelle *X*_*j*_ in *C*_*i*_:

$$X_{j}=(x_{1}\;x_{2}\;\cdots \;x_{k}\;\cdots \;x_{K}),$$ (3)

Among:

*X*_*j*_ is a set of vectors, representing the solution in the *j*-th sub-film in *C*_*i*_;

*x*_*k*_ represents the *k*-th dimension of the current solution.

In order to be more intuitive, we can directly express *X*_*j*_ in *C*_*i*_:

$$C_{i}=[\begin{array}{c} X_{1}\\ X_{2}\\ \vdots \\ X_{j}\\ \vdots \\ X_{J} \end{array}]={\left[\begin{array}{cccccc} x_{1}^{1} & x_{2}^{1} & \cdots & x_{k}^{1} & \cdots & x_{K}^{1}\\ x_{1}^{2} & x_{2}^{2} & \cdots & x_{k}^{2} & \cdots & x_{K}^{2}\\ \vdots & \vdots & \ddots & \vdots & \ddots & \vdots \\ x_{1}^{j} & x_{2}^{j} & \cdots & x_{k}^{j} & \cdots & x_{K}^{j}\\ \vdots & \vdots & \ddots & \vdots & \ddots & \vdots \\ x_{1}^{J} & x_{2}^{J} & \cdots & x_{k}^{J} & \cdots & x_{K}^{J} \end{array}\right]}_{i}$$ (4)

Among:

$x_{k}^{j}$ represents the *k*-th dimension variable of the solution in the *j*-th sub-film;

*J* represents the number of sub-membranes in the cell *C*_*i*_, and *J* = *K*+1.

Due to differences in cell organs, the nuclear membrane is the core control unit of the cell, directing each organelle membrane to process only one parameter, and the remaining parameters approach the optimal solution to increase the local search efficiency of the algorithm. The update rules for the parameters in the sub-membrane are as follows:

When *j*≠*J*:

$$x_{k}^{j}=\{\begin{array}{cc} rand\times (x_{k}^{best}-x_{k}^{j})+x_{k}^{j} & j=k\\ x_{k}^{best}+HSP\times (rand-0.5)\times (ub_{k}-lb_{k}) & j\neq K \end{array},$$ (5)

When *j* = *J*:

$$\begin{array}{l} X_{J}=[x_{1}^{J}\;x_{2}^{J}\;\cdots \;x_{k}^{J}\;\cdots \;x_{K}^{J}]\\ (x_{1}^{J}=x_{1}^{1},x_{2}^{J}=x_{2}^{2},\cdots ,x_{k}^{J}=x_{k}^{k},\cdots ,x_{K}^{J}=x_{K}^{J-1}), \end{array}$$ (6)

Among:

$x_{k}^{best}$ represents the *k*-th variable of the optimal solution in the cell *C*_*i*_;

*rand* represents a random number between 0–1;

*ub*_*k*_ represents the upper bound of the *k*-th dimension variable;

*lb*_*k*_ represents the lower bound of the *k*-th dimension variable;

The MSA abstracts missense mutations in the synthesis of new proteins in cell life activities by setting *rand* random numbers. Most missense mutations are directly or indirectly caused by affecting the folding, assembly, and transport of these proteins. Protein misfolding can lead to two situations: one is that the number of proteins that are correctly folded and transported has reduced, resulting in loss of function; the other is that misfolding can abnormally gain function. The MSA develops a search space by abstractly mimicking the way of missense mutations in the synthesis of proteins by cells.

#### 2.2.4 Protein activity model

*HSP* stands for the role of heat shock protein contained in the single-cell system. In the process of cell secretion of proteins, under the action of *HSP*, some genes are expressed under normal conditions, and some are expressed in large quantities under elevated temperature or other stress conditions to protect cells and reduce abnormal environmental damage. *α* represents the influence of temperature on *HSP*, which gradually increases with the number of iterations, while *HSP* gradually decreases from the initial value. The MSA uses this cell physiological characteristic to gradually converge the sub-membrane optimization result to the vicinity of the optimal solution with the number of iterations to obtain a more accurate local optimization. Here *Q* represents the activity constant of the enzyme in the cell, which is an empirical value, and the enzyme activity affects the accuracy of the algorithm optimization. The expressions of *HSP* and *α* are as follows:

$$\alpha =Q\times \ln\frac{s}{S}$$ (7)

$$HSP=-\frac{e^{\alpha }-1}{e^{\alpha }+1}$$ (8)

Among:

*Q* is an adjustable parameter;

*s* represents the current number of iterations;

*S* represents the maximum number of iterations.

**Fig 4:**
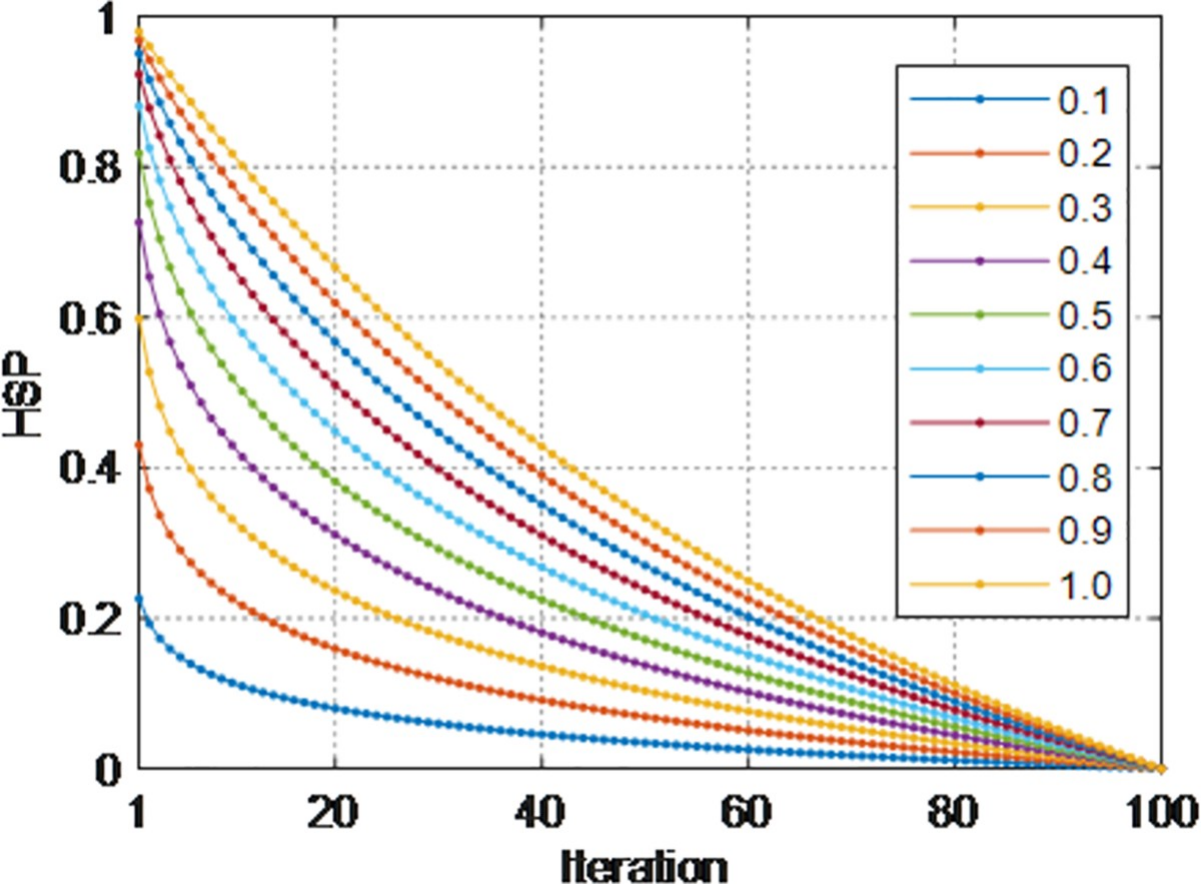
Variation curve of *HSP* with iteration under different values of *Q*.

*HSP* abstracts the gene expression effect of single-cell synthetic protein. Compared with other optimization algorithms, the *Q* parameters provided by MSA can be adjusted by users. As can be seen from the above Fig 4, the larger the value of *Q*, the greater the change of *HSP* with the step size. For the MSA, the search range of *C*_*i*_ is larger, and its ability to jump out of the local optimal solution is stronger, but the optimization accuracy will be lower; when the *Q* value is smaller, the change of *HSP* with the step size is smaller. For MSA, the search range of *C*_*i*_ is smaller, and its ability to jump out of the local optimal solution will decrease, but the optimization accuracy will increase. In this paper, we provide two empirical values. When unimodal benchmark functions and multi-modal benchmark functions, *Q* is set to 0.015 to increase the accuracy of the solution. In composite benchmark functions, *Q* is set to 0.6 to increase the cell search range to avoid falling into a local optimal solution. Users can also set their own values according to actual problems to obtain the optimal performance of MSA for different problems.

When the above update rule exceeds the parameter range, we assign the boundary to $x_{k}^{j}$. At this time, the update rule is as follows:

$$x_{k}^{j}=\{\begin{array}{c} \min(x_{k}^{j},ub_{k})\\ \max(x_{k}^{j},lb_{k}) \end{array}$$ (9)

Among:

$x_{k}^{j}$ represents the *k*-th dimensional variable of the solution in the *j*-th sub-membrane;

*ub*_*k*_ represents the upper bound of the *k*-th dimension variable;

*lb*_*k*_ represents the lower bound of the *k*-th dimension variable.

The pseudo code of MSA local search process is as follows:

Pseudo code:

    *for* each membrane *C*_*i*_ indexed by *i*

        *for* each membrane *X*_*j*_ indexed by *j*

            *for* each membrane *x*_*k*_ indexed by *k*

            *r*2 = *random*[0,1]

            *if*: *j*≠*J*

                *if*: *j* = *k*

                    $x_{k}^{j}=r2\times (x_{k}^{best}-x_{k}^{j})+x_{k}^{j}$

                *else*

                    $x_{k}^{j}=x_{k}^{best}+HSP\times (rand-0.5)\times (ub_{k}-lb_{k})$

                *end if*

            *else*

                $x_{k}^{J}=x_{k}^{k}$

            *end if*

            *end for*

       *end for*

    *end for*

In the MSA local search process, the *HSP* gradually decreases from the initial value as the number of iterations increases. The MSA uses this cellular physiological characteristic to gradually converge the optimization result to the vicinity of the optimal solution with the number of iterations to obtain a more accurate local optimization. As MSA decreases with *HSP*, its local search will gradually converge to the vicinity of the current optimal solution.

#### 2.2.5 Intercellular information exchange model

**Fig 5:**
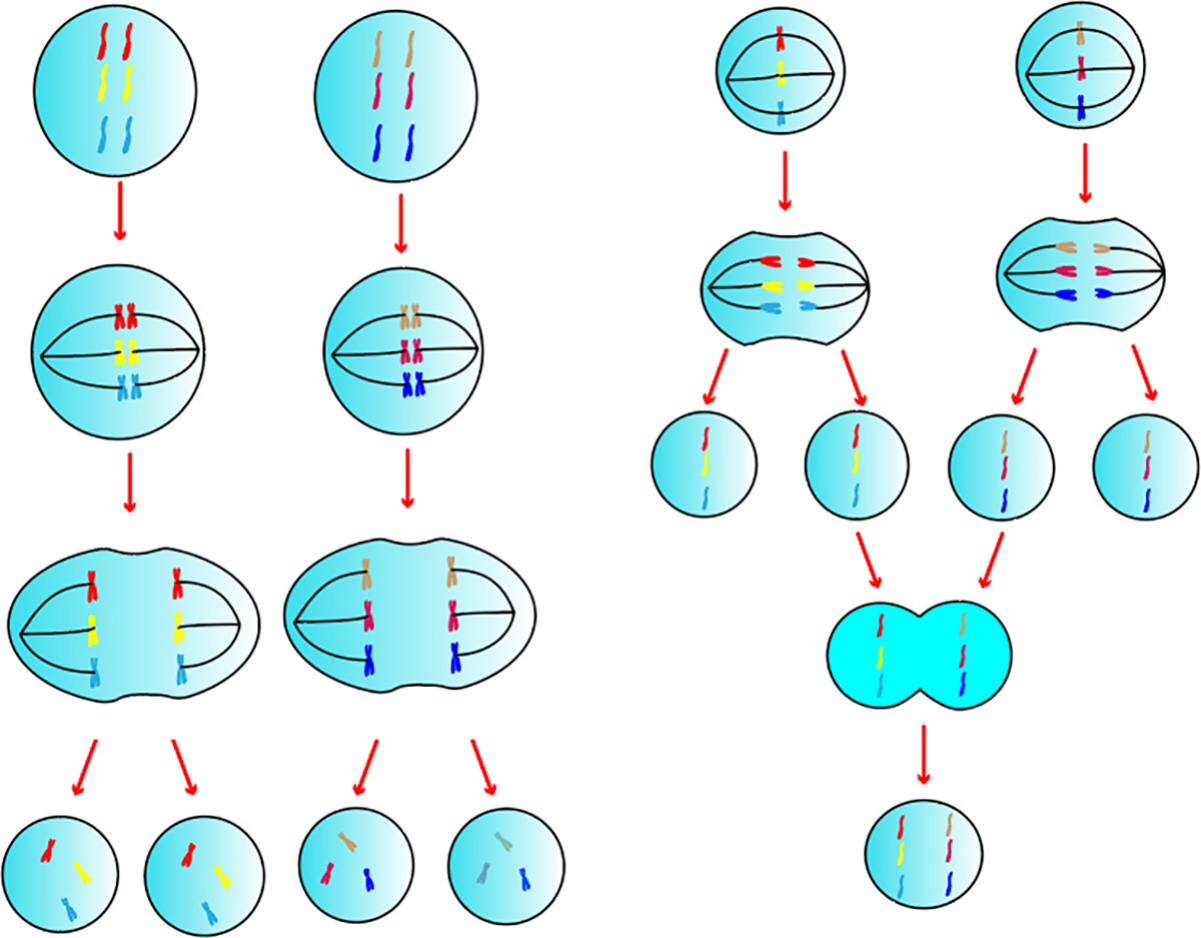
Meiotic fusion model.

As shown in Fig 5, cells exchange genetic material through meiosis and sexual reproduction. We introduce the concepts of cell meiosis and sexual reproduction as the global optimization scheme of the algorithm. The significance of meiosis is that it cannot only effectively obtain the genetic material of the parents, maintain the genetic stability of the offspring, but also increase more variation and ensure biodiversity.

In the process of biological cell reproduction in nature, the optimal individual always has the priority of reproduction. We take advantage of this feature to use the cell containing the optimal solution in the population as the male parent for division and fusion, and the remaining cells as the female parent for fusion. As the algorithm continues to iterate, since the population’s optimal child cell membrane individual exists as the parent, other cells will continue to evolve toward the optimal individual through reproductive behavior. We set the threshold of 0.75. While ensuring evolution, it will retain certain mutations and improve the ability of MSA to jump out of local optimal solutions. The advantages of this design will be reflected in the subsequent optimization of complex functions.

The intercellular fusion rules are updated as follows:

$$C_{i}^{best}=\{\begin{array}{c} rand\times (C_{best}-C_{i}^{best})+C_{i}^{best}\;(rand\geq 0.75)\\ C_{best}+rand\times (C_{best}-C_{i}^{best})\;(rand<0.75) \end{array}$$ (10)

Among:

$C_{i}^{best}$ represents the optimal solution in the *i*-th single cell membrane system in the MSA;

*C*_*best*_ represents the global optimal solution of the MSA;

*rand* represents a random number between 0–1.

The pseudo code of the information exchange model between cells is as follows:

Pseudo code:

*for* each membrane *C*_*i*_ indexed by *i*

*r*1 = *random*[0,1]

*if*: *r*1≥0.75

$C_{i}^{best}=r1\times (C_{best}-C_{i}^{best})+C_{i}^{best}$

*else*

$C_{i}^{best}=C_{best}+r1\times (C_{best}-C_{i}^{best})$

*end if*

*end for*

The global search strategy of MSA seeks the best in the space between the global optimal solution and the local optimal solution and does not converge with the number of iterations.

## 3. Results and discussion

In this section, the MSA is based on 19 benchmark functions. The first 13 benchmark functions are classic functions used by most meta-heuristic algorithm researchers [19–24]. Although these benchmark functions are relatively simple, they are representative and convenient for comparison with other algorithms due to their wide application. In the test, the MSA. The 13 benchmark functions can be divided into two categories, unimodal functions, and multimodal functions.

These benchmark functions are listed in the following Tables 1 and 2. In the table, *dim* represents the depth of the function, *Range* represents the boundary of the function search space, and *f*_min_ is the best value. In addition, the other test platforms we chose came from the six complex functions of the CEC meeting. These composite functions are composite functions generated after displacement, rotation, and combination of classic functions. The detailed functions of compound functions can be introduced in the CEC-2005 paper [25].

**Table 1:** Unimodal benchmark functions.

Function	Dim	Range	*f*_min_
$F_{1}(x)=\sum_{i=1}^{n}x_{i}^{2}$	15	[–100,100]	0
$F_{2}(x)=\sum_{i=1}^{n}|x_{i}|+\prod_{i=1}^{n}|x_{i}|$	15	[–10,10]	0
$F_{3}(x)=\sum_{i=1}^{n}{\left(\sum_{j-1}^{i}x_{j}\right)}^{2}$	15	[–100,100]	0
$F_{4}(x)=\max_{i}\{|x_{i}|,1\leq i\leq n\}$	15	[–100,100]	0
$F_{5}(x)=\sum_{i=1}^{n‐1}[100{\left(x_{i+1}-x_{i}^{2}\right)}^{2}+{\left(x_{i}-1\right)}^{2}]$	15	[–30,30]	0
$F_{6}(x)=\sum_{i=1}^{n}([x_{i}+0.5]{)}^{2}$	15	[–100,100]	0
$F_{7}(x)=\sum_{i=1}^{n}ix_{i}^{4}+random[0,1]$	15	[-1.28,1.28]	0

**Table 2:** Multi-modal benchmark functions.

Function	Dim	Range	*f*_min_
$F_{8}(x)=\sum_{i=1}^{n}-x_{i}^{}\;\sin(\sqrt{\left|x_{i}\right|})$	15	[–500,500]	0
$F_{9}(x)=\sum_{i=1}^{n}[x_{i}^{2}-10\;\cos(2\pi x_{i})+10]$	15	[-5.12,5.12]	0
$F_{10}(x)=-20\;\exp(-0.2\sqrt{\frac{1}{n}\sum_{i=1}^{n}x_{i}^{2}})-\exp(\frac{1}{n}\sum_{i=1}^{n}\cos(2\pi x_{i}))+20+e$	15	[–32,32]	0
$F_{11}(x)=\frac{1}{4000}\sum_{i=1}^{n}x_{i}^{2}-\prod_{i=1}^{n}\cos(\frac{x_{i}}{\sqrt{i}})+1$	15	[–600,600]	0
$\begin{array}{l} F_{12}(x)=\frac{\pi }{n}\{10\;\sin(\pi y_{1})+\\ \sum_{i=1}^{n}{\left(y_{i}-1\right)}^{2}[1+10\;\sin^{2}(\pi y_{i+1})]+{\left(y_{n}-1\right)}^{2}\}\\ +\sum_{i=1}^{n}u(x_{i},10,100,4)\\ y_{i}=1+\frac{x_{i}+1}{4}\\ u(x_{i},a,k,m)=\left\{ \begin{array}{c} k{\left(x_{i}-a\right)}^{m}\;x_{i}>a\\ \;0\;-a<x_{i}<a\\ k{\left(-x_{i}-a\right)}^{m}\;x_{i}>-a \end{array}\right. \end{array}$	15	[–50,50]	0
$\begin{array}{l} F_{13}(x)=0.1\{\sin^{2}(3\pi x_{1})+\\ \sum_{i=1}^{n}{\left(x_{i}-1\right)}^{2}[1+\sin^{2}(3\pi x_{i}+1)]+{\left(x_{n}-1\right)}^{2}[1+\sin^{2}(2\pi x_{n})]\}\\ +\sum_{i=1}^{n}u(x_{i},5,100,4) \end{array}$	15	[–50,50]	0

To verify the performance of the algorithm, we compare the MSA with the MVO algorithm, GWO algorithm, MFO algorithm, and ALO algorithm. These algorithms are all newly proposed meta-inspired optimization algorithms in recent years [26, 27]. They are widely used [28, 29]. The number of iterations of each algorithm on each benchmark function is 100, the number of clusters is 10, and the run is repeated 50 times, and the average and standard deviation of the results are recorded.

**Fig 6:**
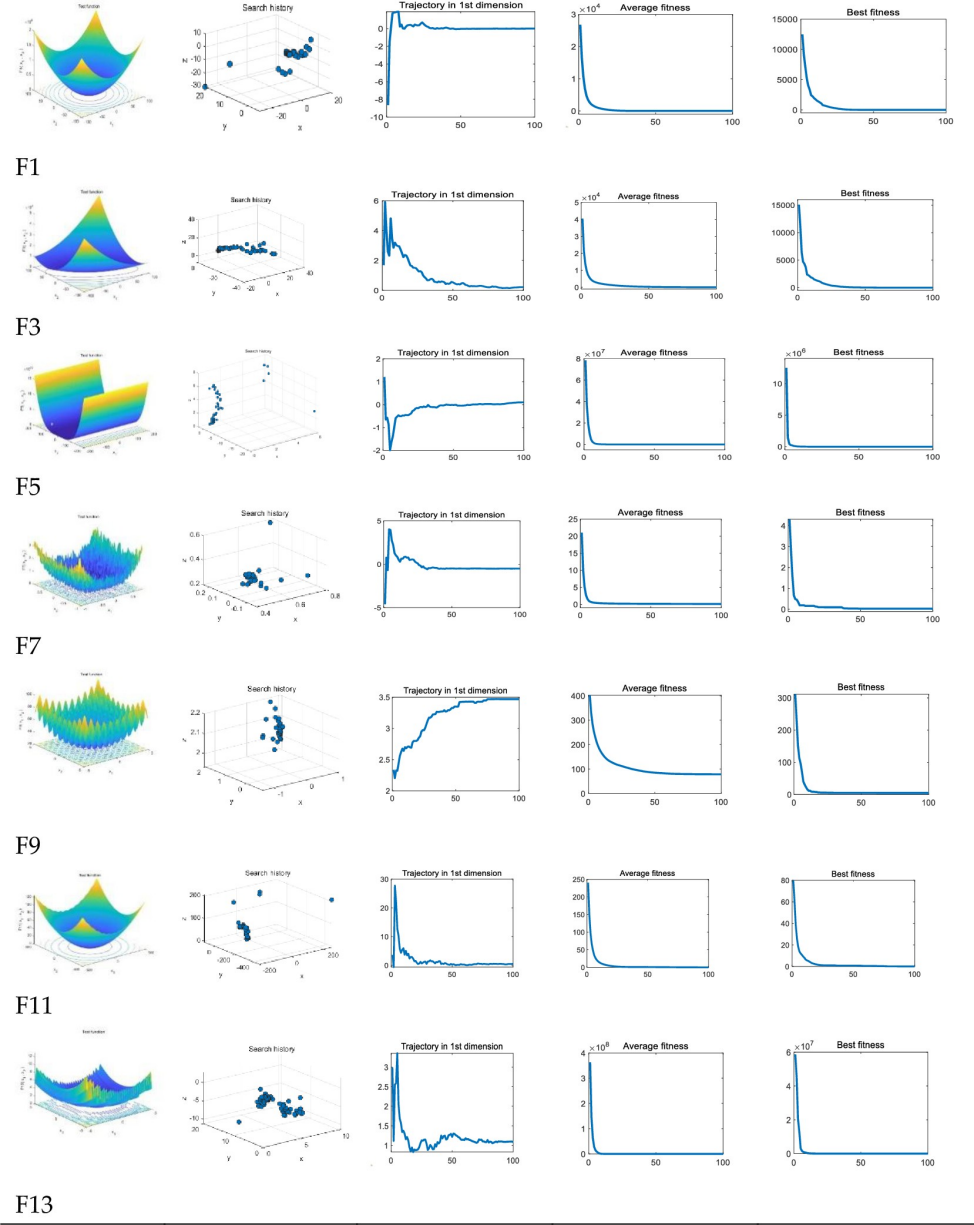
MSA search history of unimodal and multi-modal benchmark functions.

In Fig 6, the first column represents the three-dimensional image of the tested function; the second column represents the three-dimensional position change of the optimal individual’s parameters during the optimization process of the MSA; The third column is the first-dimensional parameter change of the optimal individual in the MSA; the fourth column represents the fitness of the optimal individual in the MSA swarm optimization algorithm changes with the iteration step; Since the algorithm is run 50 times repeatedly, the aforementioned parameters are all averaged 50 times; the fifth column represents the fitness of the optimal individual obtained by the MSA in 50 repetitions.

The MSA can provide competitive results. From the second column of the three-dimensional map in above Fig 6, we can find that the MSA searches around the best point at the beginning, and continues to converge to the best point with iterations. This is due to the design of the MSA imitating the organelle, which makes a single cell Individuals have the ability to seek advantages and avoid disadvantages so that areas of possible optimal solutions can be quickly found in the cell population.

The fourth column of average fitness reflects that the algorithm can quickly converge. The best individual in the population is used as the parent, and other individuals are fused with the best individual to obtain the best individual solution information. This design can accelerate the convergence in the process of unimodal function optimization, thereby increasing the convergence speed of a single iteration; due to the number of local optimal solutions of unimodal function and multimodal function is small, to improve the optimization accuracy, we have adjusted the parameters down in the peak and multi-peak functions, which will explore the space with smaller steps in the later iteration of the MSA to obtain more accurate optimization results. In above Fig 6, the third column reflects the changing trend of the first-dimensional parameters of the MSA. It can be seen from Fig 6. that although the starting point is not good, there are large-scale mutations in the initial parameters of the MSA, and the mutation amplitude is gradually reduced with the number of iterations. The design is conducive to the exploration of the entire region in the early stage of the algorithm, and the targeted development is carried out after determining the region where the optimal solution may exist in the later stage.

From Table 3, it can be found that the MSA has better results than the other four algorithms in the F1 and F6 functions, and good results can also be obtained in the F2 and F7 functions. It is worth mentioning that the unimodal function reflects the ability of algorithm benchmark development, which shows that the MSA is excellent in this respect.

**Table 3:** Results of unimodal benchmark functions.

F	MSO		MVO		GWO		ALO		MFO	
	Ave	SD	Ave	SD	Ave	SD	Ave	SD	Ave	SD
F1	8.7495e-04	3.2273e-04	10.2313	5.2546	0.0533	0.0571	4.8967e+03	2.1150e+03	1.6096e+03	2.2590e+03
F2	0.3055	0.6260	14.0613	18.0058	0.0386	0.0168	49.6471	14.3902	21.2112e+04	10.8184
F3	74.4146	116.8520	973.8978	438.9209	86.9044	77.6368	1.1807e+04	6.5782e+03	1.2120e+04	5.8021e+03
F4	2.8612	3.0346	10.1195	8.8488	0.8728	0.4719	34.9484	8.5643	62.1272	11.0091
F5	1.1816e+03	3.9738e+03	3.5711e+03	7.5622e+03	19.4736	15.6933	2.3029e+06	2.1211e+06	2.3087e+06	9.6379e+06
F6	7.6043e-04	2.6048e-04	8.9862	3.5897	1.7697	0.5089	4.9878e+03	2.6931e+03	2.0847e+03	2.3933e+03
F7	0.0863	0.0475	0.0752	0.0373	0.0254	0.0144	1.9431	1.0283	1.9560	3.0985

Compared with the unimodal function, the multi-modal function has many local optimal values, and the number of them increases exponentially with the dimension. These functions are suitable for the exploration ability of the detection algorithm. As can be seen from the above Table 4, the MSA has strong adaptability to multi-modal benchmark functions. From the one-dimensional image in the third column, we can know that the data has multiple vertical changes, which reflects the ability of the MSA to jump out of the local optimal solution. In the first row and third column of Fig 6, the algorithm has a large mutation in the middle of the iteration. This mid-term ultra-amplitude mutation ability benefits from the abstraction of the concept of cell meiosis and sexual reproduction by the MSA. Using a mutation strategy to maintain the diversity of algorithm solutions is an effective method [30], which allows MSA to effectively retain the optimal individual information during the iteration process, maintain the stability of the algorithm optimization, and increase more mutations to improve the algorithm of resisting local optimal solution capabilities.

**Table 4:** Results of multi-modal benchmark functions.

F	MSO		MVO		GWO		ALO		MFO	
	Ave	SD	Ave	SD	Ave	SD	Ave	SD	Ave	SD
F8	4.7462e+03	302.4690	3.7632e+03	408.1472	2.9235e+03	625.6863	2.8628e+03	477.2798	4.0958e+03	458.9983
F9	78.3987	38.0840	119.9696	44.5255	123.6363	40.8783	1.3525	0.8352	23.6579	7.0332
F10	3.5607	2.6745	6.3676	6.8130	0.0715	0.0444	15.4090	2.7799	15.5257	3.8678
F11	0.0218	0.0181	1.0751	0.0587	0.2089	0.1104	43.9563	19.4804	17.3948	21.6136
F12	8.6309	5.4757	3.9959	2.7825	0.6128	0.4580	1.4070e+06	3.1366e+06	1.5243e+06	3.3789e+06
F13	1.3219	4.4374	1.1764	1.0255	1.1853	0.4562	4.7577e+06	7.005e+06	1.1410e+07	1.8578e+07

According to the above Table 4, the MSA achieves better results than other algorithms on the F11 function and also achieved competitive results in the F10 and F13 functions. It reflects that MSA has a strong global exploration ability while retaining the ability to prevent falling into local optimal solutions.

**Fig 7:**
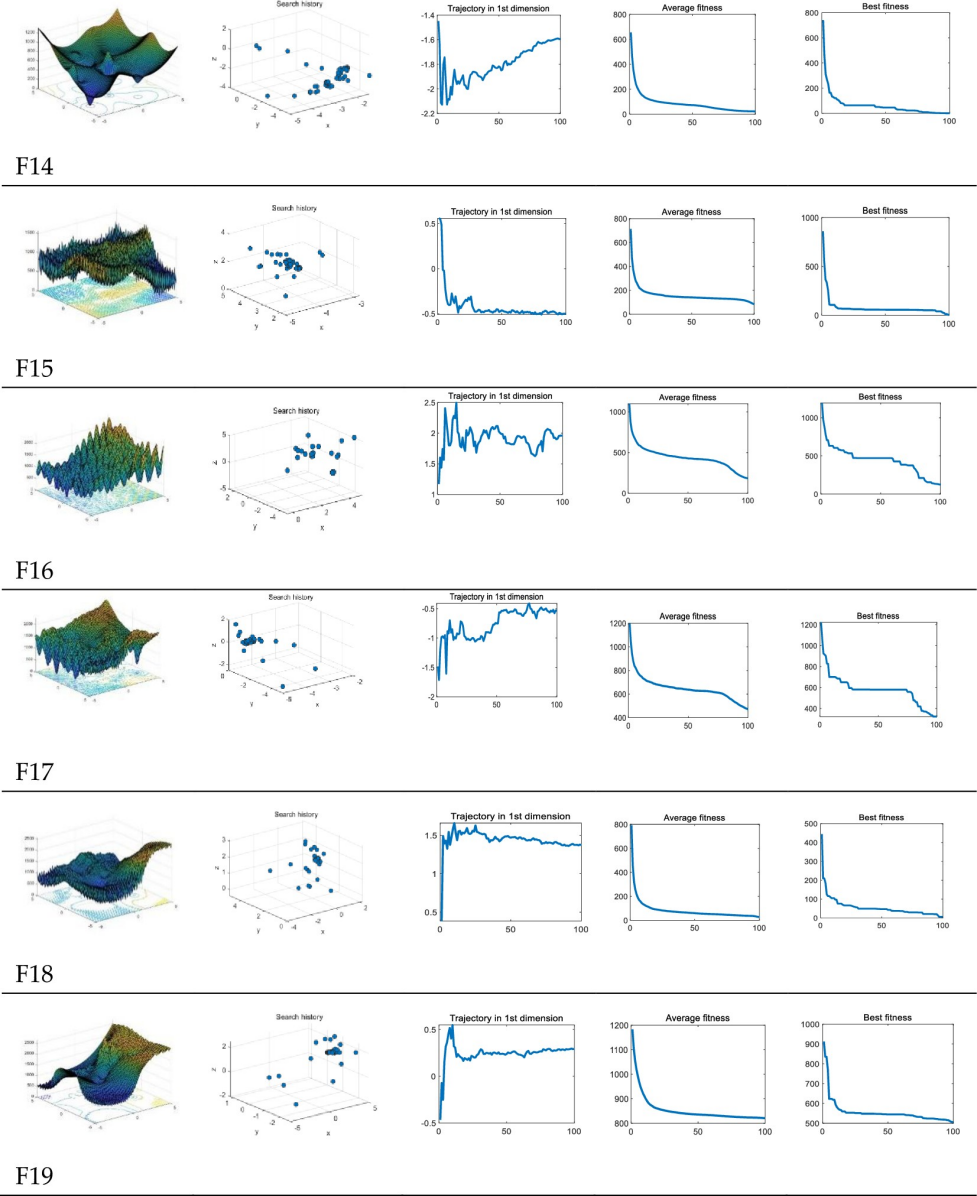
MSA search history of composite benchmark functions.

As shown in Table 5, The composite benchmark function is composed of multiple groups of different functions, which is a very challenging test for the optimization algorithm. It can be seen from the above Fig 7 that the local search space of MSA gradually shrinks with the step size, which is similar to other group optimization algorithms. This design can help the algorithm to conduct extensive exploration in the global stage in the early stage and can help the algorithm to search in the region in the later stage.

**Table 5:** Composite benchmark functions.

Function	Dim	Range	*f*_min_
$\begin{array}{l} F_{14}(CF1)\\ f_{1},f_{2},f_{3},\ldots ,f_{10}=\mathrm{Sphere}\;\mathrm{function}\\ \left[\sigma_{1},\sigma_{2},\sigma_{3},\ldots ,\sigma_{10}]=[1,1,1,\ldots 1\right]\\ \left[\lambda_{1},\lambda_{2},\lambda_{3},\ldots \lambda_{10}]=[5/100,5/100,5/100,\ldots 5/100\right] \end{array}$	15	[–5,5]	0
F15(CF2)f1,f2,f3,…,f10=Griewank'sfunction[σ1,σ2,σ3,…,σ10]=[1,1,1,…1][λ1,λ2,λ3,…λ10]=[5/100,5/100,5/100,…5/100]	15	[–5,5]	0
F16(CF3)f1,f2,f3,…,f10=Griewank'sfunction[σ1,σ2,σ3,…,σ10]=[1,1,1,…1][λ1,λ2,λ3,…λ10]=[1,1,1,…1]	15	[–5,5]	0
F17(CF4)f1,f2=Ackley'sfunctionf3,f4=Rastrigin'sfunctionf5,f6=Weierstrassfunctionf7,f8=Ackley'sfunctionf9,f10=Spherefunction[σ1,σ2,σ3,…,σ10]=[1,1,1,…1][λ1,λ2,λ3,…λ10]=[5/32,5/32,1,1,5/0.5,5/100,5/100,5/100,5/100]	15	[–5,5]	0
F18(CF5)f1,f2=Rastrigin'sfunctionf3,f4=Weierstrassfunctionf5,f6=Griewank'sfunctionf7,f8=Ackley'sfunctionf9,f10=Spherefunction[σ1,σ2,σ3,…,σ10]=[1,1,1,…1][λ1,λ2,λ3,…λ10]=[1/5,1/5,5/0.5,5/0.5,5/100,5/100,5/32,5/32,5/100,5/100]	15	[–5,5]	0
F19(CF6)f1,f2=Rastrigin'sfunctionf3,f4=Weierstrassfunctionf5,f6=Griewank'sfunctionf7,f8=Ackley'sfunctionf9,f10=Spherefunction[σ1,σ2,σ3,…,σ10]=[0.1,0.2,0.3,0.4,0.5,0.6,0.7,0.8,0.9,1.0][λ1,λ2,λ3,…λ10]=[0.1×1/5,0.2×1/5,0.3×5/0.5,0.4×5/0.5,0.5×5/100,0.6×5/100,0.7×5/32,0.8×5/32,0.9×5/100,1×5/100]	15	[–5,5]	0

However, limited by this design, the algorithm is easy to fall into the local optimal solution in the later stage. But for the MSA, the method of information fusion does not change with the step size as the inter-group communication rule, and its mutation ability always exists, which ensures that MSA has a strong ability in the optimization of super-complex composite functions. When the MSA falls into the local optimal solution, the search mechanism of the MSA gives it the ability to escape the current space. When testing the multi-modal function above, the parameter variation in the middle of the iteration has been reflected. The test of the composite benchmark function highlights the role of this ability. Facts have proved that this mechanism not only effectively maintains the stability of excellent solutions but also can increase more search and development capabilities.

The composite benchmark function used is usually a very challenging test platform for meta-heuristic algorithms. It can detect the detection and development performance of the algorithm at the same time. Since this function has a large number of local optimal solutions, the ability of the function to avoid locally optimal solutions can be tested. According to the above Table 6, MSA has achieved very competitive results while maintaining high computational stability.

**Table 6:** Results of composite benchmark functions.

F	MSO		MVO		GWO		ALO		MFO	
	Ave	SD	Ave	SD	Ave	SD	Ave	SD	Ave	SD
F14	22.1549	54.5508	161.0825	152.4570	232.0816	157.6860	947.0148	164.2346	203.8868	122.6354
F15	84.5699	77.4758	279.0466	137.0534	359.6516	136.7675	1.0774e+03	145.1174	242.2932	128.6801
F16	183.5834	37.7175	502.9318	167.7558	489.4202	162.6323	1.4215e+03	164.0751	454.9907	119.4715
F17	473.0368	135.4676	673.1883	115.3223	741.2412	152.7680	1.3797e+03	91.6158	717.0409	103.9583
F18	27.7213	35.4273	313.1734	256.5552	338.5649	252.2804	1.2528e+03	181.9147	194.3647	171.7738
F19	820.9068	134.9647	892.2839	101.1834	897.1799	14.2607	1.3389e+03	97.1536	893.0512	101.1834

The test provided in this article proves that the MSA has excellent optimization capabilities, but it cannot be considered that the MSA is better than other test algorithms. With reference to NFL theory, different algorithms show different capabilities in solving various problems. Only in the specific benchmarks provided in this article, the MSA embodies very competitive advantages in some aspects, which makes it used to solve practical problems.

## 4. MSA for classical engineering problems

In this section, two constrained engineering problems are adopted: welded beam design, pressure vessel design. These problems have equality and inequality constraints. We provide constraint conditions by increasing the penalty factor [38].

### 4.1 Welded beam design

**Fig 8:**
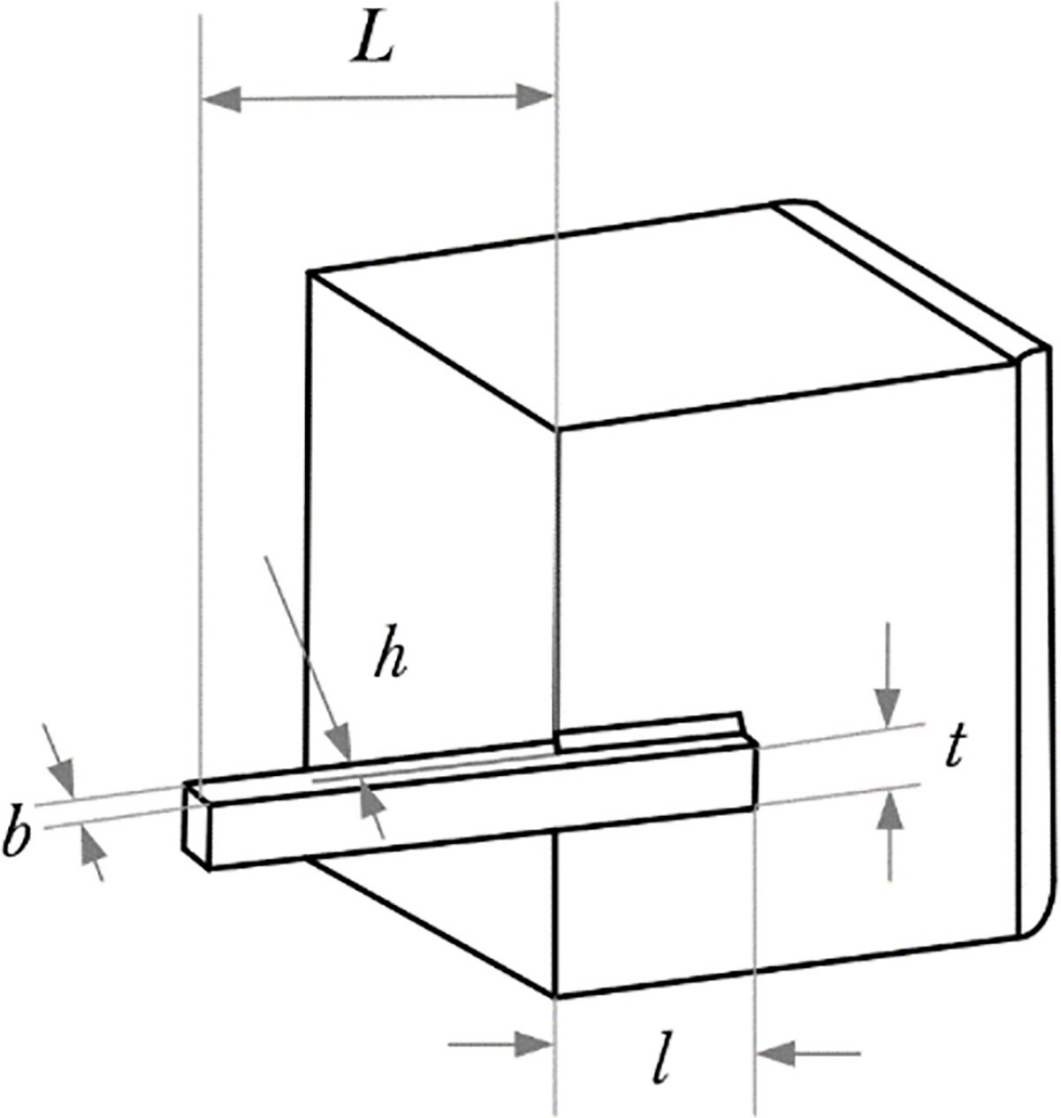
Design parameters of the welded beam design problem.

The design of welded beams is a classic engineering problem, which aims to reduce the manufacturing cost of the welded beam structure. The welded beam structure shown in Fig 8 consists of beam A and the welding required to be connected to part B.

We realize the optimization of the problem by controlling the four structures of the welded beam design structure: the thickness of the weld (*h*), the length of the clamping rod (*l*), the height of the rod (*t*), and the thickness of the rod (*b*).

The constraints and problems of the welded beam design are shown in Table 7. The literature [33] uses the MVO algorithm, the literature [31] uses the MFO algorithm, and the literature [32] eta. used successive linear approximation methods to solve such problems. The comparison results have shown in Table 8, and the MSA has found the lowest cost design.

**Table 7:** Constraint condition of welded beam.

Welded beam design	
Consider:	
	$\vec{x}=[x_{1},x_{2},x_{3},x_{4}]=[h,l,t,b]$
Minimize:	
	$\begin{array}{l} f(\vec{x})=1.10471x_{1}^{2}x_{2}+\\ 0.04811x_{3}x_{4}(14.0+x_{2}) \end{array}$
Subject to the following constraints:	
	$g_{1}(\vec{x})=\tau (\vec{x})-\tau_{\max}\leq 0$ $g_{2}(\vec{x})=\sigma (\vec{x})-\sigma_{\max}\leq 0$ $g_{3}(\vec{x})=x_{1}-x_{4}\leq 0$ $\begin{array}{l} g_{4}(\vec{x})=1.10471x_{1}^{2}+\\ 0.04811x_{3}x_{4}(14.0+x_{2})-5.0\leq 0 \end{array}$ $g_{5}(\vec{x})=0.125-x_{1}\leq 0$ $g_{6}(\vec{x})=\delta (\vec{x})-\delta_{\max}\leq 0$ $g_{7}(\vec{x})=P-P_{C}(\vec{x})\leq 0$
Variable range:	
	0.1≤*x*_1_≤2 0.1≤*x*_2_≤10 0.1≤*x*_3_≤10 0.1≤*x*_1_≤2
Where the other auxiliary formula:	
	$\tau (\vec{x})=\sqrt{({\left(\tau^{\prime }\right)}^{2}+{\left(\tau^{\prime \prime }\right)}^{2})+\frac{2\tau^{\prime }\tau^{\prime \prime }x_{2}}{2R}},\tau^{\prime }=\frac{p}{\sqrt{2}x_{1}x_{2}}$ $\tau^{\prime \prime }=\frac{MR}{J},M=P(L+\frac{x_{2}}{2}),$ $R=\sqrt{{\left(\frac{x_{1}+x_{3}}{2}\right)}^{2}+\frac{x_{2}^{2}}{4}}$ $J=2\{\sqrt{2}x_{1}x_{2}[\frac{x_{2}^{2}}{12}+{\left(\frac{x_{1}+x_{3}}{2}\right)}^{2}]\},\;\sigma (\vec{x})=\frac{6PL}{x_{4}x_{3}^{2}}$ $\delta (\vec{x})=\frac{4PL^{3}}{Ex_{4}x_{3}^{3}},P_{c}(\vec{x})=\frac{4.013\sqrt{\frac{EGx_{3}^{2}x_{4}^{6}}{36}}}{L^{2}}(1-\frac{x^{3}}{2L}\sqrt{\frac{E}{4G}})$
Related parameters:	
	$P=6000lb,L=14in.,\delta_{\max}=0.25in.,E=30\times 10^{6}\;psi$ $G=12\times 10^{6}\;psi,\tau_{\max}=13600psi,\sigma_{\max}=30000psi$

**Table 8:** Comparison results of the welded beam.

Algorithm	Optimum variables				Optimum cost
	*h*	*l*	*t*	*b*	
MSA	0.2055	3.4756	9.0365	0.2057	1.7252
MFO [31]	0.2035	3.4430	9.2302	0.2123	1.7325
GWO [32]	0.2056	3.4783	9.0368	0.2057	1.7262
MVO [33]	0.2056	3.4721	9.0409	0.2057	1.7254
ALO	0.2757	5.0746	8.9974	0.3020	2.9198
GA [34]	0.1641	4.0325	10.0000	0.2236	1.8739
HS [35]	0.2442	6.2231	8.2915	0.2443	2.3807
Radom [36]	0.4575	4.7313	5.0853	0.6600	4.1185

### 4.2 Pressure vessel design problem

**Fig 9:**
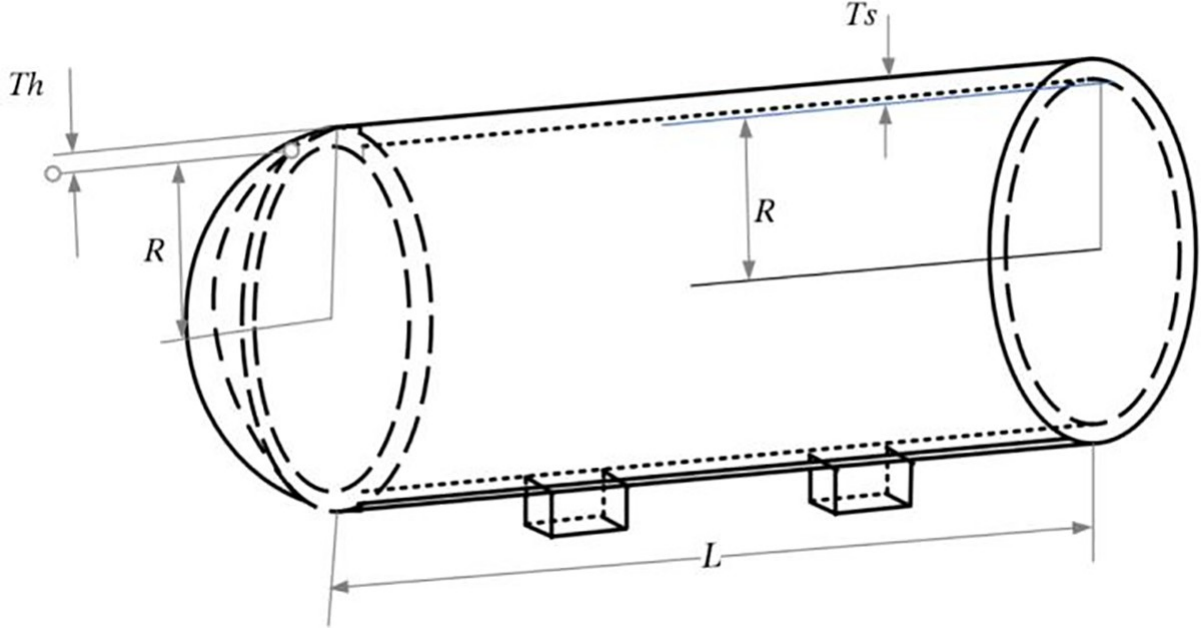
Pressure vessel.

The purpose of this problem is to minimize the cost of material, molding, and welding of the cylindrical container. As shown in Fig 9, the head of the container is hemispherical, and both ends are designed with lids. The container design needs to consider four variables: Shell thickness (*T*_*s*_); Head thickness (*T*_*h*_); Inner diameter (*R*); Excluding the length of the cylindrical section of the head (*L*).

The constraints and problems of the pressure vessel design are shown in Table 9. Many researchers have used different algorithms to solve this design problem, such as MFO [31], MVO [33], GA [34], HS [35], etc. Table 10 gives a comparison of the best solutions so far obtained through MSA and other algorithms, which have previously been reported in the literature on pressure vessel design problems, and the MSA has found the lowest cost design.

**Table 9:** Constraint condition of pressure vessel.

Pressure	
Consider:	
	$\vec{x}=[x_{1},x_{2},x_{3},x_{4}]=[T_{s},T_{h},R,L]$
Minimize:	
	$f(\vec{x})=0.6224x_{1}x_{3}x_{4}+1.7781x_{2}x_{3}^{2}+3.1661x_{1}^{2}x_{4}+19.84x_{1}^{2}x_{3}$
Subject to the following constraints:	
	$g_{1}(\vec{x})=-x_{1}+0.0193\;x_{3}\leq 0$ $g_{2}(\vec{x})=-x_{3}+0.00954\;x_{3}\leq 0$ $g_{3}(\vec{x})=-\pi x_{3}^{2}x_{4}-\frac{4}{3}\pi x_{3}^{3}+1296000\leq 0$ $g_{4}(\vec{x})=x_{4}-240\leq 0$
Variable range:	
	0≤*x*_1_≤99 0≤*x*_2_≤99 10≤*x*_3_≤200 10≤*x*_1_≤200

**Table 10:** Comparison results of pressure vessel.

Algorithm	Optimum variables				Optimum cost
	*T*_*s*_	*T*_*h*_	*R*	*L*	
MSA	0.7783	0.3851	40.3283	199.9008	5887.7052
MFO [31]	0.8352	0.4098	43.5786	152.2152	6055.6378
GWO [32]	0.8125	0.4345	42.0891	176.7587	6051.5639
MVO [33]	0.8457	0.4185	43.8162	156.3816	6011.5148
PSO [37]	0.8125	0.4375	42.0984	176.6365	6059.7143
GA [34]	0.7523	0.3995	40.4525	198.0026	5890.3279
HS [35]	1.0995	0.9065	44.4563	179.6588	6550.0230

## 5. Conclusions and future work

The meta-heuristic algorithm proved to be an effective method to solve optimization problems. With the continuous development of artificial intelligence, in the field of optimization, the objective function of nonlinear engineering design optimization problems often contains many local optimal solutions. However, designers are always concerned about finding the global optimal solution. To solve this problem, this paper proposes a multi-membrane search algorithm (MSA) inspired by cell behavior. And through 19 benchmark functions to test the MSA, and compared with the emerging meta-inspired optimization algorithms MVO, GWO, MFO, and ALO in recent years, the following conclusions are drawn.

1. MSA has efficient convergence capabilities on unimodal functions and multimodal functions, which shows that MSA has better global optimization capabilities and faster search efficiency than other tested algorithms.
2. By introducing the concepts of cell meiosis and sexual reproduction, the algorithm maintains stability while increasing the diversity of candidate solutions. This makes MSA better than other tested algorithms in the composite function, which shows that MSA can achieve a good dynamic balance between exploration and development. Compared with other tested algorithms, MSA is less likely to fall into a local optimal solution.
3. The MSA is also competitive in solving classic engineering problems. A result of 1.7252 was obtained in the design problem of welded beams, and a result of 5887.7052 was obtained in the design problem of pressure vessels. Compared with other algorithms, MSA found a lower cost design, which proved that the MSA is effective in practical applications. Other researchers can try to use the MSA to solve similar engineering problems.

For future work, we will implement the MSA through FPGA and apply it to the field of parallel computing.

Pseudo code:

*Create random Multi-membrane M*

*Initialize HSP*, *α*, *and S*.

*while*(*s*≤*S*)

    *Evaluate the fitness of all C*_*i*_

    *Update C*_*best*_ *and* $C_{i}^{best}$

    *for each membrane C*_*i*_ *indexed by i*

        *r*1 = *random*[0,1]

        *if*: *r*1≥0.75

            $C_{i}^{best}=r1\times (C_{best}-C_{i}^{best})+C_{i}^{best}$

        *else*

            $C_{i}^{best}=C_{best}+r1\times (C_{best}-C_{i}^{best})$

        *end if*

    *end for*

        *for each membrane C*_*i*_ *indexed by i*

            *for each membrane X*_*j*_*indexed by j*

                *for each membrane x*_*k*_ *indexed by k*

                *r*2 = *random*[0,1]

                *if*: *j*≠*J*

                    *if*: *j = k*

                        $x_{k}^{j}=r2\times (x_{k}^{best}-x_{k}^{j})+x_{k}^{j}$

                *else*

                        $x_{k}^{j}=x_{k}^{best}+HSP\times (rand-0.5)\times (ub_{k}-lb_{k})$

                    *end if*

                *else*

                    $x_{k}^{J}=x_{k}^{k}$

                *end if*

                *end for*

            *end for*

        *end for*

    *Update HSP indexed by Eq (8)*

    *Update α indexed by Eq (7)*

    *Update S indexed by s* = *s*+1

*end while*

## Supporting information

S1 Data (MAT)

S2 Data (MAT)
